# Polarization‐Improved Bidirectional‐Pump Atomic Magnetometer Based on Spin‐Decoupled Metasurface

**DOI:** 10.1002/advs.202509028

**Published:** 2025-07-06

**Authors:** Shuo Sun, Jiahao Zhang, Rongtong Zhu, Huanyu Zhou, Tianshi Cheng, Liang Chen, Jin Li

**Affiliations:** ^1^ School of Instrumentation and Optoelectronic Engineering Beihang University Beijing 100191 China; ^2^ College of Optical and Electronic Technology China Jiliang University Hangzhou 310018 China; ^3^ National Institute of Extremely‐Weak Magnetic Field Infrastructure Hangzhou 31005 China; ^4^ Beihang Hangzhou Innovation Institute Hangzhou 310052 China

**Keywords:** atomic magnetometer, bidirectional pump, metasurface, optically pumped, spin‐decoupled

## Abstract

The optically pumped atomic magnetometer (OPAM) has emerged as an advanced instrument for magnetic field detection. However, the performance of conventional OPAMs is constrained by factors such as non‐uniform atomic polarization and insufficient precision in system polarization control. In this study, a bidirectional‐pump OPAM based on spin‐decoupled metasurface is presented and experimentally demonstrated. By designing a metasurface structure with a predefined phase distribution, the system generates two circularly polarized (CP) lights with distinct chirality and one linearly polarized (LP) light. Two beams of the CP light are directed in opposite directions along both sides of the cell, which effectively mitigates the polarization gradient within the cell and enhances the stability of the system. Additionally, the use of zero‐order LP light as the probe light for the system enhances energy utilization efficiency while simplifying the system architecture. The experimental results demonstrate that the OPAM based on metasurface successfully realizes precise control of atomic spin polarization. The proposed OPAM is capable of performing high‐precision magnetic field measurements, and the sensitivity has been improved to 1.85 pT/Hz^1/2^. This research not only advances the application of metasurfaces in quantum precision measurement but also offers a novel approach for enhancing the performance of atomic magnetometers.

## Graphical abstract

The polarization‐improved bidirectional‐pump atomic magnetometer based on spin‐decoupled metasurface has been experimentally demonstrated. The system, based on a metasurface, successfully realizes precise control of atomic spin polarization. The research not only offers a novel technique for designing high‐performance optically pumped atomic magnetometers but also paves the way for the application of metasurfaces in quantum precision measurement.

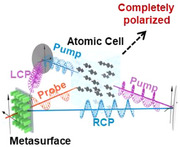

## 1 Introduction

The atomic magnetometers, as highly sensitive magnetic field measurement instruments, hold significant application potential in geomagnetic field detection, medical imaging, and quantum information.^[^ ^1^, ^2^, ^3^ ^]^ Among these, the optically pumped atomic magnetometer (OPAM) has emerged as one of the current research focal points due to its wide dynamic range and non‐contact measurement characteristics.^[^ ^4^, ^5^, ^6^ ^]^ Its operating principle is grounded in the Larmor precession of a polarized ensemble of alkali‐metal under an external magnetic field. Precise measurement of the magnetic field is accomplished through the application of optical pump and optical probe techniques.^[^ ^7^, ^8^ ^]^ The light absorption detection method and the optical polarization rotation angle detection method represent two frequently employed technical approaches. The optical absorption detection method enables the measurement of magnetic field intensity by assessing the impact of the precession direction of polarized atomic spins, induced by an external magnetic field, on the absorption of pump light. This method features a simple system structure and ease of implementation. However, its sensitivity is constrained by optical path noise and detector performance.^[^ ^9^, ^10^, ^11^ ^]^ In contrast, the optical polarization rotation angle detection method relies on the Faraday rotation effect of the polarized alkali‐metal ensemble, which causes linearly polarized (LP) light to generate an optical rotation angle. By detecting the rotation angle of the polarization plane, the magnetic field intensity can be inversely calculated. Due to its insensitivity to light intensity jitter and the ability to substantially enhance the signal‐to‐noise ratio via polarization modulation technology, this method is regarded as having greater sensitivity potential and broader application prospects.^[^ ^12^, ^13^, ^14^, ^15^ ^]^ However, in the conventional approach to detecting optical polarization rotation angle, the pump light is subject to atomic absorption, which leads to a reduced overall atomic polarizability and the undesired polarization gradient along the direction of the optical pump.^[^ ^16^, ^17^, ^18^ ^]^ Additionally, this method encounters challenges such as inefficient polarization modulation, intricate optical setups, and susceptibility to interference from environmental magnetic fields.^[^ ^19^, ^20^, ^21^ ^]^ These limitations, to some extent, constrain its applicability in complex magnetic field scenarios and high‐precision measurement contexts.

Metasurface, as a novel 2D structure enabled by advancements in artificial electromagnetic material technology, offers a promising method for enhancing the polarization uniformity of OPAMs and simplifying system complexity.^[^ ^22^, ^23^, ^24^, ^25^ ^]^ This is attributed to its unique capability to manipulate the light field with high‐precision. The metasurface consists of structural units at the subwavelength scale. By precisely engineering the structural units, it is possible to achieve efficient manipulation of the amplitude, phase, and polarization state of the optical field.^[^ ^26^, ^27^, ^28^, ^29^ ^]^ Specifically, the metasurface is capable of freely customizing and generating different polarization states. Moreover, it can also be utilized to enhance the polarization efficiency of atoms during the optical pump.^[^ ^30^, ^31^, ^32^ ^]^ Meanwhile, this method can also be employed to modulate the polarization state of the probe light, thereby enhancing the contrast and signal‐to‐noise ratio of the optical detection signal. Furthermore, the metasurface exhibits the characteristics of being lightweight and easily integrable, thereby offering promising potential for the miniaturization of OPAMs. However, currently, research on OPAMs based on metasurfaces remains in the exploratory stage. Metasurface primarily functions to perform polarization modulation of atomic pump light or balanced polarimetry.^[^ ^33^, ^34^ ^]^ The designed metasurfaces usually generate a single polarization state and are influenced by zero‐order light, thereby leading to suboptimal conversion results.^[^ ^35^, ^36^, ^37^ ^]^ Moreover, it is frequently restricted to use at only one end of the pump and probe process, whereas the other end continues to rely on conventional optical components. Consequently, the enhancement in both performance and portability of the OPAMs remains constrained.^[^ ^38^, ^39^, ^40^, ^41^, ^42^ ^]^ Therefore, designing a metasurface with multi‐polarization modulation functionality holds great significance for enhancing atomic polarizability. Moreover, this approach can fulfill all polarization states required by OPAMs, thereby facilitating their integration and high‐performance development.

Here, we proposed the polarization‐improved bidirectional‐pump OPAM based on a spin‐decoupled metasurface. Through the innovative design and optimization of the metasurface, high‐quality left circularly polarized (LCP), right circularly polarized (RCP), and LP lights for the OPAMs were successfully generated. By employing gradient phase technology, the decoupling of various polarization states was successfully realized. Specifically, LCP and RCP lights were incident from the front and rear of the atomic cell, respectively, thereby achieving bidirectional pumping with enhanced polarization characteristics. This incident mode design effectively mitigates the polarization gradient in the cell, thereby achieving an improved signal‐to‐noise ratio and detection sensitivity, while also enhancing the stability of the system. In addition, the zero‐order LP light of the metasurface is innovatively employed as the probe source, thereby achieving efficient energy utilization and a compact design while enhancing the precision of optical probing. The experimental results demonstrate that the OPAM system constructed with spin‐decoupled metasurface exhibits superior detection performance within the magnetic field range up to 20 000 nT and shows strong applicability in complex magnetic field environments. The sensitivity has been enhanced from 4.88 pT/Hz^1/2^ with the conventional method to 1.85 pT/Hz^1/2^ at 10 Hz. Meanwhile, all polarization modulation functions within the single metasurface integrated system have been successfully implemented. This not only enhances the conversion accuracy but also substantially diminishes the system's complexity. The research not only offers a novel technique for designing high‐performance OPAMs but also paves the way for the application of metasurfaces in areas such as quantum sensing and precision measurement.

## 2 Results and Discussion

### 2.1 Design OPAM Based on Spin‐Decoupled Metasurface

**Figure 1:**
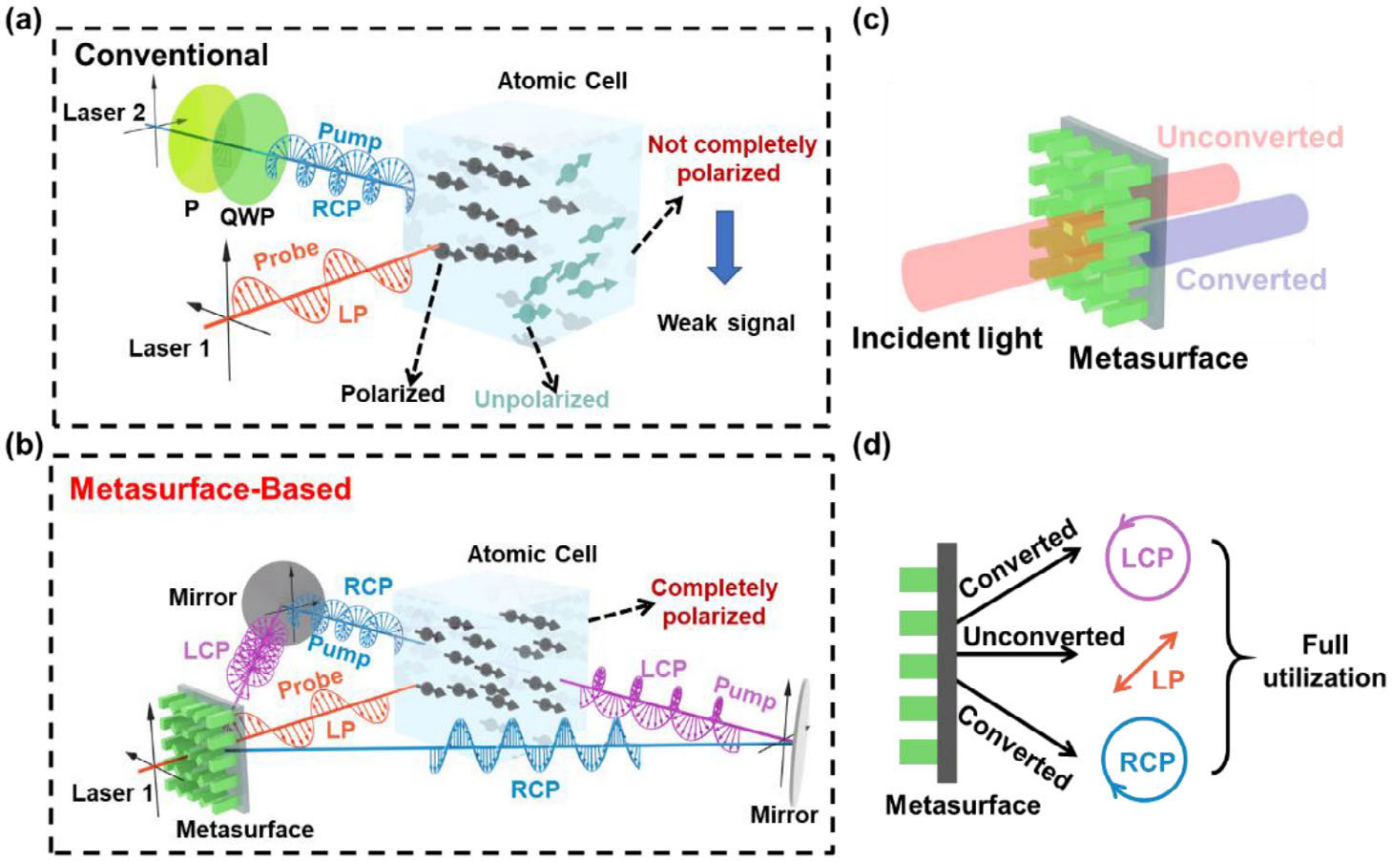
Comparison between conventional optically pumped atomic magnetometer (OPAM) and the proposed OPAM based on spin‐decoupled metasurface. a) Schematic of incomplete atomic polarization causing a weak signal in the conventional OPAM. b) Schematic of the proposed bidirectional‐pump OPAM based on spin‐decoupled metasurface. c) Schematic of converted light and unconverted zero‐order light in metasurface optical field modulation. d) Schematic of the designed metasurface generating left circularly polarized (LCP), right circularly polarized (RCP), and linearly polarized (LP) light with full utilization.

The response of the spin polarization of alkali‐metal ensembles to an external magnetic field serves as the fundamental principle for OPAMs. In the conventional OPAM system employing the optical polarization rotation angle detection method, as depicted in **Figure** **1** **a**, LP light produced by Laser 1 is utilized for probing, while the combination of Laser 2, a polarizer (P), and a quarter‐wave plate (QWP) is employed to generate circularly polarized light for atomic pumping. However, due to atomic absorption, the atoms located at the front of the cell absorb a significant portion of the pump light. Consequently, the atoms at the rear fail to acquire sufficient energy, leading to reduced polarizability. This phenomenon gives rise to an atomic polarization gradient, which induces polarization inhomogeneity within the cell. Such inhomogeneity weakens the system response and thereby degrades the overall performance and stability of the system. The proposed bidirectional polarization OPAM, which is based on a spin‐decoupled metasurface, employs the metasurface as the exclusive polarization conversion element, as depicted in Figure 1b. LP light generated by a laser is directly incident on the metasurface. After modulation by the metasurface, LCP and RCP lights are produced. Influenced by the phase gradient, these two circularly polarized beams are separated. This approach not only effectively maintains the degree of polarization of the circularly polarized light, but also enables the light to be directed into the OPAM from both the front and rear ends of the cell via reflectors. The evolution of the atomic spin polarization under the influence of an external magnetic field can be described by the following Bloch equation:

(1) $$\frac{dP}{dt}=\gamma B\times P+R_{op}\left(s\hat{z}-P\right)-R_{rel}P$$

Where *s* represents the spin angular momentum of the z‐axis pump beam. The spin angular momenta of RCP and LCP lights along the positive z‐axis are +1 and ‐1 (σ^+^ and σ^−^), respectively, in units of electron spin angular momentum ℏ. Considering the interaction of circularly polarized light with opposite rotation directions within the system relative to the positive and negative directions of the z‐axis, we can define that σ^+^ corresponds to the positive direction of the z‐axis while σ^−^ corresponds to the negative direction of the z‐axis. Consequently, the product of $s\hat{z}$ remains consistently positive (The detailed theoretical model can be found in Note S1, Supporting Information). Therefore, the pumping polarization effect of the counter‐propagating RCP and LCP lights on the alkali‐metal atomic ensemble is consistent. Meanwhile, it is precisely the bidirectional incidence that enables the optimal configuration for the utilization of pump light energy and the enhancement of polarization. It ensures that atoms at both the front and rear ends of the cell are polarized with uniform energy, thereby effectively minimizing the polarization gradient (The numerical simulation for the spin polarization of atomics is shown in Figure S1, Supporting Information). As a result, complete polarization of the alkali‐metal ensemble can be achieved, which in turn improves the performance and stability of the OPAMs.

Due to the characteristics of metasurface materials and the processing accuracy, metasurfaces frequently exhibit unmodulated zero‐order light. This untransformed portion of the light often represents a significant challenge that requires suppression or filtering in light field modulation, as illustrated in Figure 1c. However, given that the designed spin‐decoupled metasurface is capable of deflecting the converted circularly polarized light at a specific angle for emission, the zero‐order LP light emitting vertically can serve as the probe light by being incident on the system. As depicted in Figure 1d, the metasurface modulation generates LCP and RCP lights, which serve as the pump lights for bidirectional pumping to achieve uniform atomic polarization. The emitted zero‐order light is utilized as the probe light, enabling efficient energy utilization and significant simplification of the system.

### 2.2 Design and Optimization for Spin‐Decoupled Metasurface

**Figure 2:**
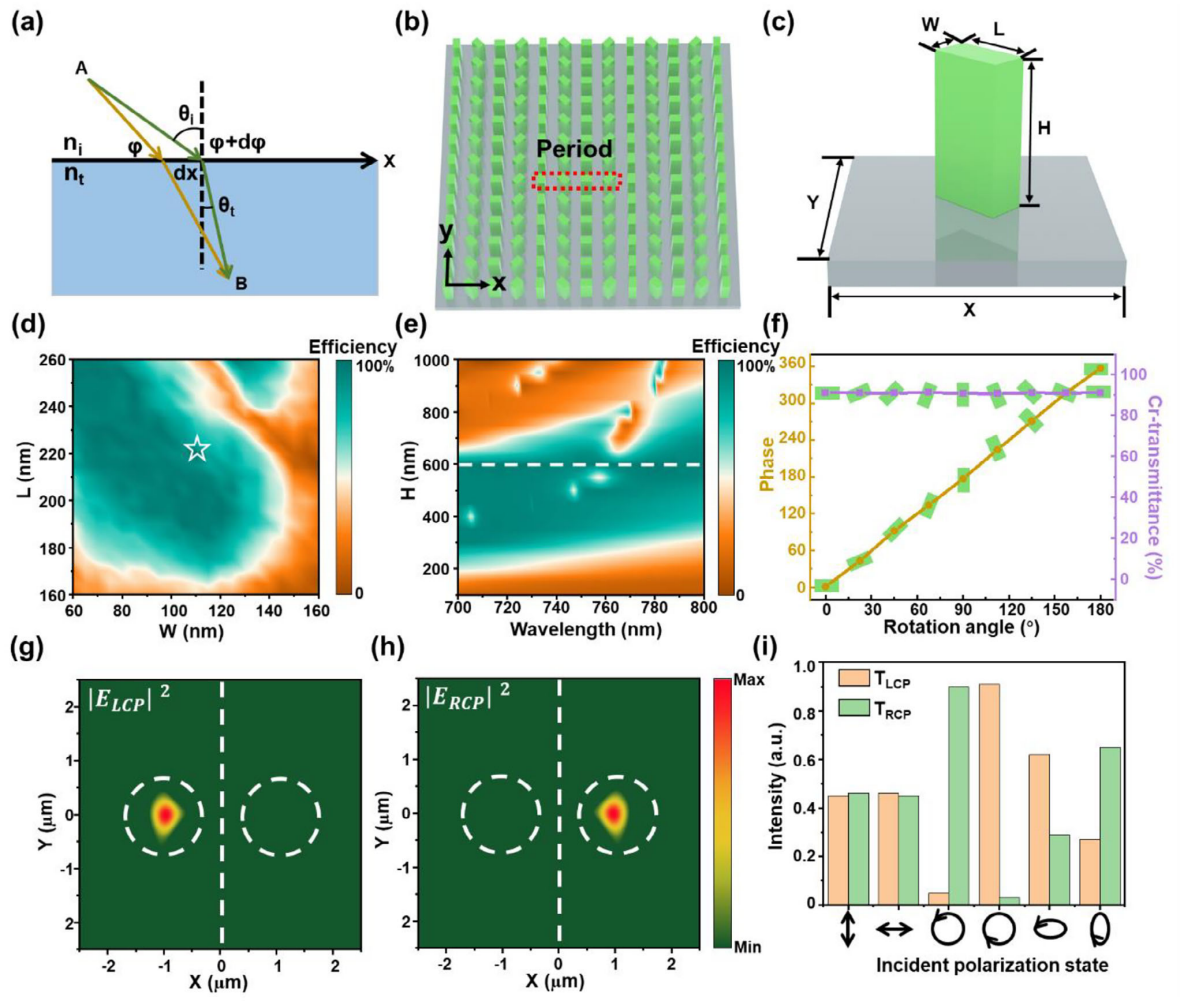
Design and simulation of spin‐decoupled metasurface. a) Schematic of anomalous refraction via abrupt phase modulation in the metasurface. b) Schematic of periodic arrangement with the metasurface phase gradient. c) Nanopillars of the metasurface with period (X, Y), height (H), length (L), and width (W). d) Cross‐polarized transmission of nanopillars with varying lengths and widths at 795 nm wavelength. e) Cross‐polarized transmission of nanopillars with varying heights across the 700–800 nm wavelength range. f) Phase and cross‐polarized transmission of nanopillars at different rotation angles. g) Left circularly polarized (LCP) intensity distribution of out‐of‐plane under linearly polarized (LP) incidence. h) Right circularly polarized (RCP) intensity distribution of out‐of‐plane under LP illumination. i) LCP and RCP intensities under incidence with different polarization states.

Design and optimize an efficient spin‐decoupled metasurface to fulfill the requirements of high‐performance OPAMs. Based on the generalized Snell's law (**Figure** **2** **a**), when a beam is incident upon the metasurface, the emergent light exhibits an anomalous refraction phenomenon caused by the abrupt phase shift at the surface. In the case of normal incidence, the refraction angle of the emergent light can be expressed as:

(2) $$\;\theta_{t}=\;\arcsin\frac{\lambda d\varphi }{2\pi dx}$$

Where λ represents the operational wavelength, and *d*φ denotes the phase gradient. The relationship between the rotation step size ▵ϑ of the metasurface unit structure and the induced phase shift *d*φ can be expressed as follows:

(3) dφ=2σ▵ϑ

The value of σ being ±1 corresponds to RCP and LCP light, respectively. Consequently, the decoupling through refraction of two circularly polarized lights with opposite chirality can be realized by designing phase gradients. As depicted in Figure 2b, four nanopillars are defined as one period, with a rotation angle difference of 45° between adjacent nanopillars. Amorphous silicon (α − Si) is employed as the material for the nanopillars, while silicon dioxide (SiO_2_) serves as the substrate. The nanopillars are arranged periodically along the x‐axis and repeated along the y‐axis, thereby enabling the refraction of circularly polarized light in the x‐z plane. As depicted in Figure 2c, each nanopillar has a height of *H*, a length of *L*, and a width of *W*. The single nanopillar periods along both the x‐axis and y‐axis are *X*  =  *Y*  =  300 *nm*.

The propagation of light through the nanopillars was simulated utilizing the Finite‐Difference Time‐Domain (FDTD) method, and the cross‐polarization transmittance was evaluated to determine the optimal size parameters for the nanopillars. The nanopillars with varying lengths and widths were selected for analysis, and the cross‐polarization transmittance distributions at the wavelength of 795 nm are presented in Figure 2d. When *L*  =  220 *nm* and *W*  =  110 *nm*, the cross‐polarization transmittance achieves its maximum value of 91.5%. Based on this size parameter, the cross‐polarization transmittance of various nanopillar heights was simulated within the incident light wavelength range from 700 to 800 nm. It was observed that when *H*  =  600 *nm*, the cross‐polarization transmittance exceeded 90% in this band, as depicted in Figure 2e. This method can be effectively applied to OPAMs utilizing different alkali‐metal atomic cells. For instance, the working wavelength for the OPAM using a potassium (K) atomic cell is 770 nm, while the working wavelength for the OPAMs using a rubidium (Rb) atomic cell is 795 nm. Rotating the nanopillars with the specified size parameters can induce distinct phases at varying rotation angles, as illustrated in Figure 2f. This phenomenon aligns with the theoretical relationship φ  =  2▵ϑ and facilitates the generation of a gradient phase. Meanwhile, nanopillars with varying rotation angles exhibit nearly identical high cross‐polarization transmittance, ensuring the uniform efficiency of the metasurface and enhancing the effectiveness of polarization modulation. When LP light is incident on the periodic structure of the metasurface composed of gradient‐phase nanopillars, the electric field intensity components of LCP and RCP in the x‐y plane of the outgoing light are extracted, as depicted in Figure 2g,h. Under the electric field intensity component of LCP light, a pronounced light spot appears on the left side, whereas no corresponding light spot is observed for the electric field intensity component of RCP light at the same position. This demonstrates that the beam on the left side corresponds to LCP light. Similarly, the light refracted to the right exhibits RCP light, thereby demonstrating that the designed metasurface can efficiently convert LP light into two distinct circularly polarized lights of opposite chirality. Altering the polarization state of the incident light and measuring the outgoing intensities of the two circularly polarized lights with opposite rotation directions, as depicted in Figure 2i. Regardless of whether the incident light is LP along the x polarization direction or the y polarization direction, the outgoing intensities of the LCP and RCP lights are essentially identical. When the polarization state of the incident light is either LCP or RCP, the emergent light exhibits only the corresponding orthogonal circular polarization state, while the circular polarization component with the same rotation direction is effectively negligible. In the case of elliptically polarized incident light, both LCP and RCP components can be emitted; their intensities differ due to the specific polarization composition of the incident elliptical polarization. The metasurface we designed and optimized is capable of effectively generating LCP and RCP lights, irrespective of the polarization state of the incident light. Furthermore, it employs a gradient phase to independently refract the two beams.

### 2.3 Fabrication and Experimental Characterization of Metasurface

**Figure 3:**
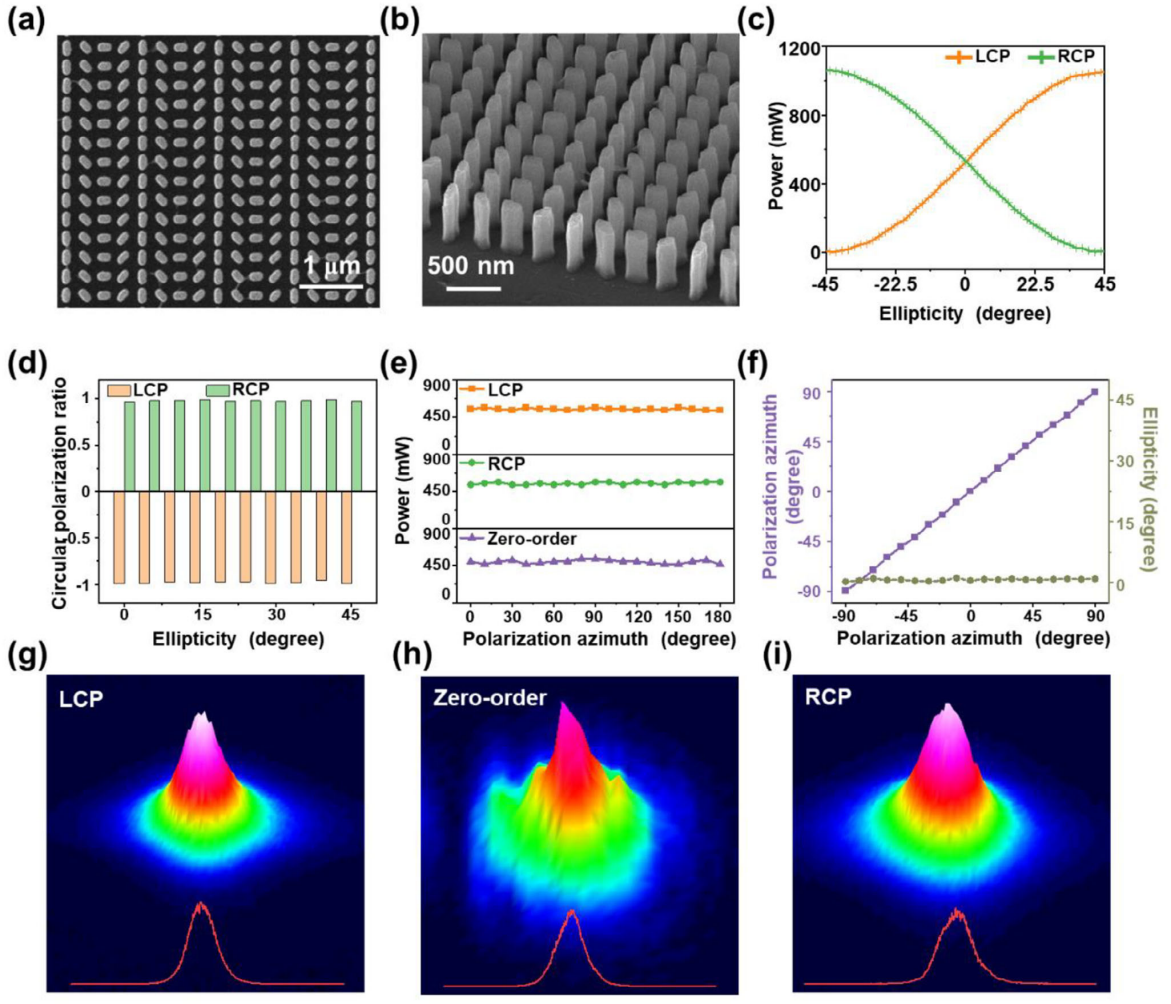
Fabrication and experimental characterization of the metasurface. a) The top‐view SEM image of the metasurface. b) The aerial view SEM image of the metasurface. c) Experimentally measured left circularly polarized (LCP) and right circularly polarized (RCP) optical power under illumination with different polarization ellipticities. d) Circular polarization ratio (experimental ellipticity normalized to 45°; −1 indicating pure LCP and 1 indicating pure RCP) under different incident polarization ellipticities. e) Optical power of output LCP, RCP, and zero‐order light under linearly polarized (LP) light incidence with varying polarization azimuth. f) Polarization azimuth and ellipticity of output zero‐order light under LP incidence with varying polarization azimuth. g) Beam quality of modulated LCP (inset: intensity profile along beam centerline). h) Beam quality of modulated zero‐order light (inset: intensity profile along beam centerline). i) Beam quality of modulated RCP (inset: intensity profile along beam centerline).

Based on the results of the design and simulation optimization, we fabricated the metasurface and characterized its optical performance. The metasurface was fabricated using lithography and etching techniques, and its morphology was characterized by SEM. As depicted in **Figure** **3** **a**, the top‐view SEM image of the metasurface demonstrates that the structure is uniformly arranged with a discernible rotation angle. However, due to limitations in processing precision, the edges and corners of the nanopillars lack clear boundaries. As depicted in Figure 3b, the aerial view of the metasurface reveals that the nanopillars exhibit non‐uniformity, with some regions appearing in an island‐like state. The fabricated metasurface exhibits a minor deviation from the designed metasurface. While this discrepancy does not compromise the functionality of the metasurface, it may lead to a slight reduction in conversion efficiency and an enhancement of zero‐order light. This represents a prevalent challenge encountered during the design and fabrication of most metasurfaces.

The measured decoupling angles for both LCP and RCP lights in the experiment were 41.5°, showing excellent agreement with the theoretical values. We measured the intensities of LCP and RCP lights on the left and right sides, respectively, under varying polarization ellipticities, as depicted in Figure 3c. The intensities of LCP and RCP lights exhibit a pattern of waxing and waning as a result of the differences in the polarization components of the incident light. The polarization states of the output light on both sides were characterized under varying ellipticities. The experimentally measured ellipticity was normalized by dividing it by 45° to define the circular polarization ratio. A value closer to −1 indicates a higher degree of LCP, while a value closer to 1 signifies a stronger RCP. As depicted in Figure 3d, under varying polarization ellipticities of the incident light, both LCP and RCP lights exhibit remarkably high circular polarization ratios. This demonstrates that the light generated through modulation is indeed standard circularly polarized light. As illustrated in Figure 3e, when the incident light is configured as LP light with varying polarization azimuths, the intensities of LCP and RCP lights remain largely consistent, indicating a stable modulation performance across different LP states. However, zero‐order light is also present. The intensity of the zero‐order light is approximately equivalent to that of LCP and RCP lights. The optical power meter is employed to measure the intensity of LCP and RCP light obtained through conversion in the exiting light. Subsequently, this intensity is normalized by dividing it by the intensity of the incident light, yielding the experimental measurement efficiency of the metasurface. Due to limitations in processing precision, the efficiency of the designed metasurface decreases from 91.5% in simulations to 60.1%. While the presence of zero‐order light does not compromise the polarization purity of the converted circularly polarized light, it results in significant energy loss. We experimentally characterized the polarization state of the zero‐order light, as depicted in Figure 3f. When illuminated with LP light at various polarization azimuths, the polarization azimuth of the outgoing light was found to be nearly identical to that of the incident light, with the ellipticity remaining very close to zero. Consequently, the polarization state of the zero‐order light closely matched that of the incident light. Therefore, when LP light generated by the laser is incident on the metasurface, not only can LCP and RCP be produced, but outgoing LP light can also exist. Moreover, these three beams of light will not overlap, thereby avoiding crosstalk effects. As illustrated in Figure 3g,i the beam quality of the three beams is evaluated. It was determined that all three beams exhibited high‐quality intensity distributions. The inset depicts the intensity distribution along the centerline of the light spot, revealing its conformity to the standard Gaussian distribution. Therefore, our experimental results have demonstrated that the designed and fabricated metasurface is capable of effectively modulating the polarization state of the incident light field. Specifically, it generates three beams with distinct polarization states: LCP, RCP, and LP. Additionally, the metasurface exhibits excellent spot quality, enabling efficient pumping and probing.

### 2.4 Construction and Performance Characterization of Designed OPAM System Based on Metasurface

**Figure 4:**
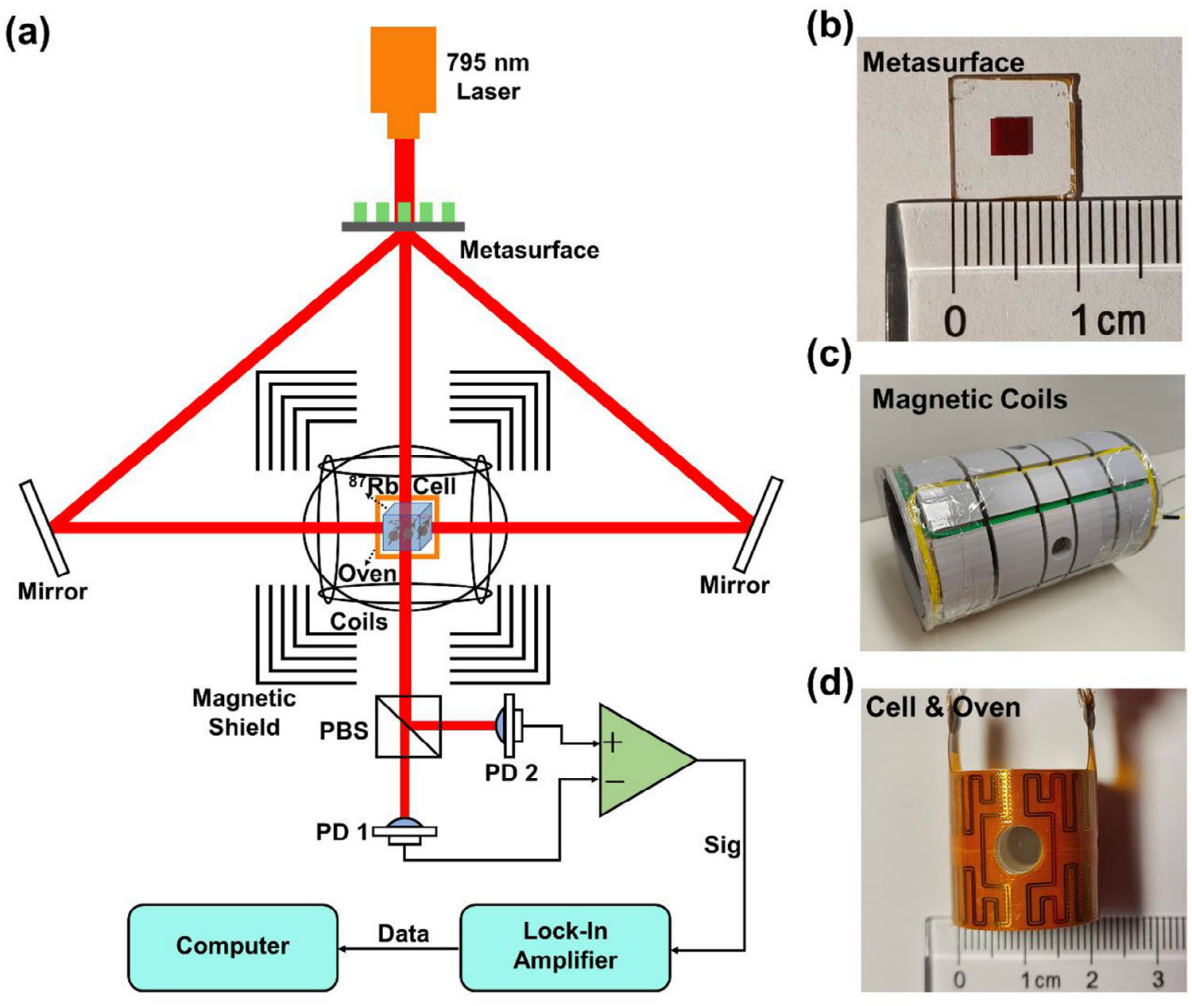
Designed an optically pumped atomic magnetometer (OPAM) system based on a spin‐decoupled metasurface. a) Schematic of bidirectional‐pump OPAM system based on spin‐decoupled metasurface. b) Photograph of the fabricated metasurface. c) Photograph of the coil holder with three orthogonal winding sets. d) Photograph of the atomic cell integrated with a heating film.

We have developed a bidirectional‐pump OPAM system based on a spin‐decoupled metasurface. As illustrated in **Figure** **4** **a**, the schematic diagram of the system is presented. The cell consists of a mixture of ^87^Rb alkali‐metal atoms and N_2_ gas for quenching. A 795 nm laser, resonant with the D1 line of ^87^Rb atoms, is employed in this system. The metasurface serves as the sole polarization modulation component. Vertically emitted LP light functions as the probe beam entering the cell, while the decoupled LCP and RCP lights are directed into the cell via the reflectors to achieve bidirectional atomic polarization. The mirror surface of the reflectors is oriented perpendicular to the horizontal plane. Upon reflection, the LCP and RCP lights undergo chiral inversion. As depicted in Figure 4b, the dimensions of the metasurface substrate are 10 mm × 10 mm, with the effective area being the central 3 mm × 3 mm region. As depicted in Figure 4c, three sets of orthogonal coils are wound on the framework to generate a stable and controllable external bias magnetic field for measurement, as well as an oscillating radio‐frequency magnetic field for exciting transverse polarization. As shown in Figure 4d, the oven and heating film surrounding the cell are positioned at the center of the coil framework. The cell is heated to 100 °C by a temperature controller to produce ^87^Rb alkali‐metal atomic vapor. The skeleton is positioned within a magnetic shielding cylinder, which consists of five layers of permalloy and serves to shield the ambient magnetic field. Bidirectional circularly polarized light, with enhanced polarization efficiency, traverses the cell to achieve effective atomic polarization while maintaining a low polarization gradient. This configuration enhances the overall stability of the system. The vertically emitted LP probe light is orthogonal to the direction of the pump light and serves to detect the precessing atomic spin polarization vector. This probe light induces a rotation angle that is proportional to the projection of the polarization vector along the direction of the probe light. Orthogonal LP beam splitting is accomplished via a polarizing beam splitter, and its intensity is measured by photodiodes PD1 and PD2. A weak oscillation signal is subsequently extracted through a differential amplifier. The oscillation component corresponding to the Larmor precession frequency is phase‐locked by the lock‐in amplifier and subsequently amplified, enabling high‐precision measurement of the magnetic field. The developed metasurface‐based OPAM system employs a single laser and a single metasurface for both pump and probe. This design not only reduces the complexity of the system but also enhances its portability through chip‐scale integration.

**Figure 5:**
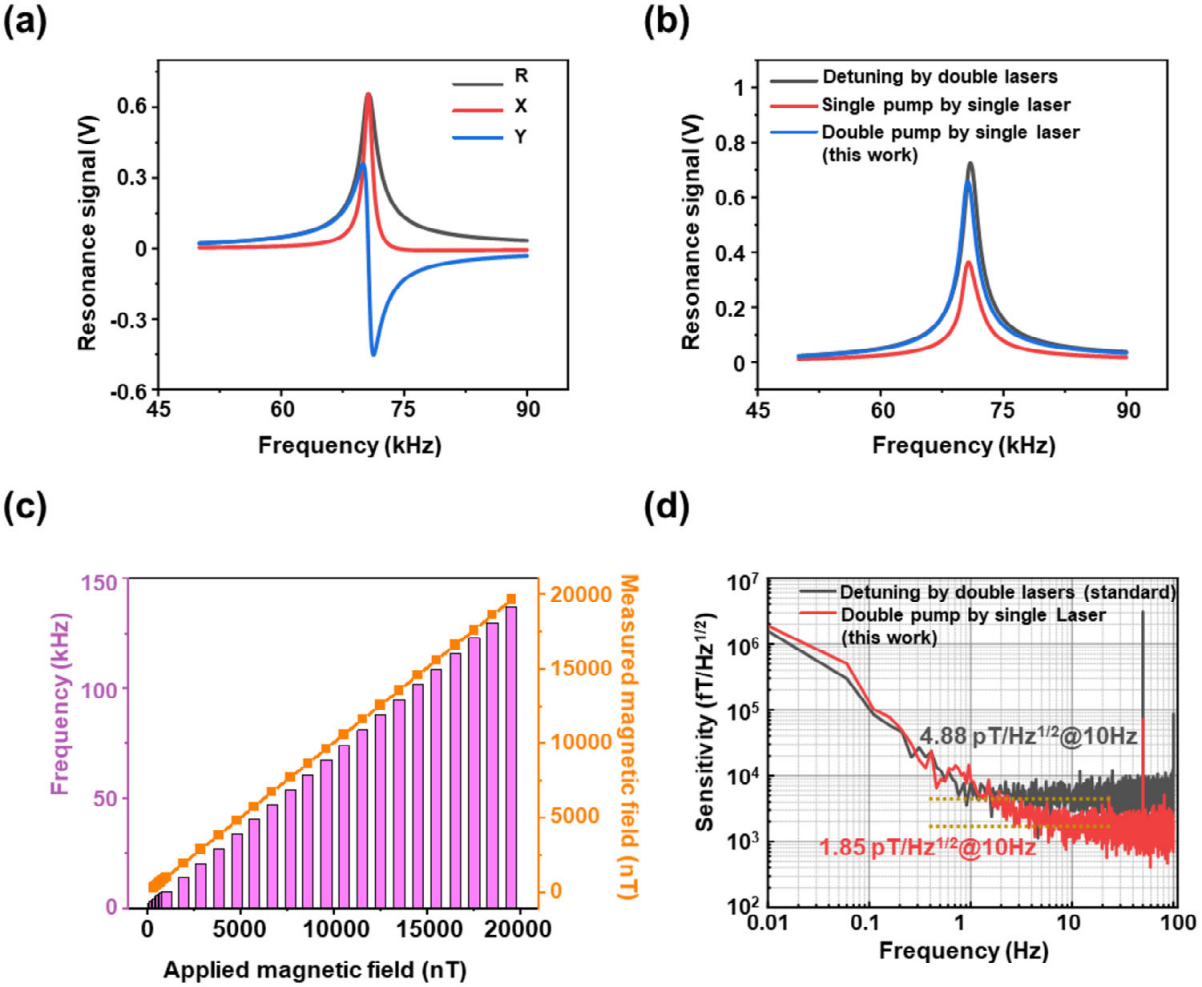
Performance characterization of the designed optically pumped atomic magnetometer (OPAM) system based on spin‐decoupled metasurface. a) Optical magnetic resonance signal under 10 000 nT external magnetic field. b) In‐phase signal intensity comparison among: conventional OPAM system with detuning by double lasers, OPAM system with single pump by single laser, and the designed OPAM system with double pump by single laser. c) Measured Larmor precession frequency and measured magnetic field under different applied magnetic field strengths (300–20000 nT). d) Sensitivity comparison between the OPAM system with detuning by double lasers and the designed OPAM system with double pumping by a single laser.

The experimental performance of the metasurface‐based OPAM system was evaluated. An external magnetic field of ≈10 000 nT was applied via the coil, and the optical magnetic resonance signal generated by the system's frequency sweep is presented in **Figure** **5** **a**. Here, R represents the modulus signal, X denotes the in‐phase signal, and Y corresponds to the quadrature signal. The high signal intensity demonstrates the stability of the system. The peak of the resonant in‐phase signal is located approximately at 70 kHz. The magnetic field magnitude calculated based on the Larmor precession frequency aligns well with the externally applied magnetic field magnitude. Consequently, the system is capable of performing accurate magnetic field measurements. Taking ^87^Rb as an example, in a conventional dual‐optical‐path OPAM, 795 and 780 nm lasers are employed as the pump and probe sources, respectively. The 780 nm laser is detuned by ≈50 GHz to minimize the interference of the probe light with atomic polarization and to reduce the absorption of the probe light by the atoms, thereby ensuring a robust detection signal. As depicted in Figure 5b, we constructed both the conventional OPAM system with detuning by double lasers and the OPAM system with a single pump by a single laser. We compared these systems with the one we designed to analyze their respective in‐phase signals. By regulating the intensity of the incident light, the optical power in each system is ensured to be stabilized at 1 mW prior to entering the cell. In each system, the cell features an outer dimension of 10 mm × 10 mm × 10 mm and an inner dimension of 5 mm × 5 mm × 5 mm. It is evident that the intensity of the resonance signal in the OPAM system with a single pump is significantly lower than that of the OPAM system with detuning by double lasers due to interference and absorption effects. Nevertheless, the system we have developed, despite utilizing a single laser, achieves a resonance signal intensity that is nearly equivalent to that of the traditional OPAM system with detuning by double lasers. This finding suggests that bidirectional pump effectively enhances uniform atomic polarization. Furthermore, this single‐frequency laser detection approach offers a novel perspective for further advancements in the system. As depicted in Figure 5c, an external magnetic field was applied within the range of 300–20 000 nT. The Larmor precession frequency was detected using the system we developed. The measured magnetic field values were found to be largely consistent with the externally applied magnetic field, thereby validating the capability of our system to accurately measure magnetic fields of varying magnitudes and operate effectively in complex magnetic environments. As depicted in Figure 5d, the sensitivity of the measurement system was evaluated to determine the minimum magnetic field it could resolve. It was found that the sensitivity of the conventional OPAM system with detuning by double lasers was 4.88 pT/Hz^1/2^ (at 10 Hz) under an external magnetic field of 10 000 nT. Under the same conditions, the sensitivity of the single pump by single laser system is 10.56 pT/Hz^1/2^ (at 10 Hz). In contrast, the OPAM system with double pump by single laser developed in this study achieved an enhanced sensitivity of 1.85 pT/Hz^1/2^ (at 10 Hz). The laser bandwidth adopted was 500 kHz. Given that the bandwidth of the pumping laser significantly influences the polarization gradient of alkali metal atoms, we subsequently employed a laser with a bandwidth of 1 MHz to replicate the experiments. Our results demonstrated that our method remains effective in suppressing the polarization gradient and enhancing the sensitivity (Note S2, Supporting Information). To investigate the influence of systematic errors on the system's sensitivity, artificial fluctuations were introduced in the heating temperature and the angle of the dual optical path, and the system's sensitivity was analyzed under the corresponding conditions. This demonstrated that the system exhibits strong stability (Note S3 and Figure S1, Supporting Information). To verify the universal applicability of the bidirectional‐pump OPAM based on the spin‐decoupled metasurface that we have designed, an OPAM based on ^39^K alkali‐metal cell was also constructed. The sensitivity of the system has been enhanced from 6.07 pT/Hz^1/2^ to 1.99 pT/Hz^1/2^ (Note S4, Supporting Information). We have successfully achieved high‐precision polarization conversion to generate high‐quality pump light by spin‐decoupled metasurface. Furthermore, we realized uniform atomic polarization via bidirectional‐pump techniques, which enables effective measurement of magnetic field strength and enhances detection sensitivity. This approach not only integrates the spin‐decoupled metasurface into atomic magnetometers in a novel application form but also offers an innovative solution for improving the performance of atomic magnetometers.

## 3 Conclusion

In conclusion, a polarization‐improved bidirectional‐pump OPAM based on spin‐decoupled metasurface has been experimentally demonstrated. By optimizing the atomic polarizability, the system performance is significantly enhanced. The metasurface was designed and optimized to effectively generate high‐quality LCP and RCP lights. Additionally, beam decoupling was achieved through the utilization of a gradient phase. The designed OPAM system employs transformed LCP and RCP lights to achieve bidirectional polarization of the atoms. This approach significantly suppresses the polarization gradient of the alkali‐metal atomic ensemble, thereby improving both the signal intensity and stability of the system. Meanwhile, employing the zero‐order LP light generated by the metasurface as the probe light enhances the energy utilization efficiency and streamlines the system configuration. Experimental performance evaluations demonstrate that the OPAM based on spin‐decoupled metasurface exhibits a broad measurement range and is capable of precisely detecting external magnetic fields reaching 20 000 nT. This system is suitable for application in geomagnetic environments, achieving an enhanced sensitivity of 1.85 pT/Hz^1/2^ at a frequency of 10 Hz. This groundbreaking solution introduces a novel technical methodology for ultra‐high‐sensitivity precise magnetic field measurement, while highlighting the substantial potential of metasurfaces in the domain of quantum sensing.

## Conflict of Interest

The authors declare no conflict of interest.

## Supporting information



Supporting Information

## Data Availability

The data that support the findings of this study are available from the corresponding author upon reasonable request.
