# Supplementary material for: Polarization‐Improved Bidirectional‐Pump Atomic Magnetometer Based on Spin‐Decoupled Metasurface
Source: Adv Sci (Weinh). 2025 Jul 6;12(37):e09028. doi: 10.1002/advs.202509028 (PMC12499377; doi:10.1002/advs.202509028)
Supplement: Supplementary file 1 — Supporting Information [file ADVS-12-e09028-s001.docx]

Supporting Information

Polarization-Improved Bidirectional-Pump Atomic Magnetometer Based on Spin-Decoupled Metasurface

Shuo Sun, Jiahao Zhang, Rongtong Zhu, Huanyu Zhou, Tianshi Cheng, Liang Chen, and Jin Li*

**Note 1.** **The theoretical model for the bidirectional-pump cell and the** **numerical simulation for** **the spin polarization of atomics.**

Optically pumped atomic magnetometers (OPAMs) utilize the alkali-metal atomic ensemble within the cell as the quantum sensing core. Under the polarization effect of the pumping light, the atomic ensemble develops a spin polarization vector. The vector undergoes the Larmor precession when subjected to an external magnetic field $B_{0}$, with its precession frequency $\omega_{L}$ being directly proportional to the modulus of the external magnetic field. The relationship is expressed as follows:

$$\begin{aligned} \omega_{L}=\gamma B_{0}\#\left( S1 \right) \end{aligned}$$

When the frequency of the applied modulation radio-frequency (RF) magnetic field equals the Larmor precession frequency $\omega_{L}$, the OPAM attains a magnetic resonant state, causing its output signal amplitude to reach an extreme value. Operating on this principle, the OPAMs achieve precision measurement of external magnetic fields by detecting the Larmor precession frequency.

The response of the alkali-metal atomic ensemble's spin polarization to external magnetic fields constitutes the theoretical foundation of OPAMs. The dynamical evolution of the spin polarization vector $\mathbf{P}=(P_{x},P_{y},P_{z})$ of the alkali-metal atomic ensemble can be phenomenologically described by the Bloch equation:

$\begin{aligned} \frac{d\mathbf{P}}{dt}=\gamma\mathbf{B}\times\mathbf{P}+R_{op}\left( s\hat{\mathbf{z}}-\mathbf{P} \right)-R_{rel}\mathbf{P}\#\left( S2 \right) \end{aligned}$Where $\gamma=7 Hz/nT$ denotes the spin gyromagnetic ratio of ⁸⁷Rb alkali-metal atoms, $\mathbf{B}=(B_{x},B_{y},B_{z})$ represents the external magnetic field vector, $R_{op}$ is the optical pumping rate, and $s$ is the spin angular momentum (SAM) of the pumping light along the z-axis. Right circularly polarized light ($\sigma^{+}$) and left circularly polarized light ($\sigma^{-}$) propagating along the z-axis carry spin angular momentum projections of $+\tilde{h}$ and $-\tilde{h}$ per photon, respectively, where $\tilde{h}$ denotes the reduced Planck constant. $R_{rel}=(R_{2},R_{2},R_{1})$ denotes the spin relaxation rate, and $R_{1}$, $R_{2}$ are the longitudinal and transverse spin relaxation rates of alkali-metal atoms, respectively.

The designed polarization-improved bidirectional-pump system employs circularly polarized lights of opposite handedness propagating along the positive and negative z-axis directions into the cell. The $\sigma^{+}$ right circularly polarized light propagates along the z-axis, while the $\sigma^{-}$ left circularly polarized light along the negative z-axis. Consequently, their spin angular momentum projections along the positive z-axis ($s\hat{\mathbf{z}}$) manifest as positive in both cases. Thus, the two counter-propagating beams induce consistent polarization effects on the alkali-metal atomic ensemble.

The alkali-metal atomic ensemble in the cell achieves depopulation through the pumping process, exhibiting macroscopic spin polarization behavior. However, due to optical absorption by the alkali-metal vapor, the intensity of the pumping light attenuates progressively with propagation distance. This attenuation causes a spin polarization gradient of the ^87^Rb atomic ensemble along the propagation axis, which severely affects the relaxation characteristics of ^87^Rb atoms and degrades both the operational stability and achievable sensitivity of OPAMs.

For the linearly polarized incident light, the variation in optical intensity $I$ can be succinctly expressed by the following equation:

$$\begin{aligned} I\left( z \right)=I\left( 0 \right)\exp\left( -n\sigma\left( \upsilon\right)z \right)\#\left( S3 \right) \end{aligned}$$

Here, $z$ denotes the propagation distance in the cell, $I(0)$ represents the intensity of the incident light, $n$ is the number density of alkali-metal atoms within the cell, and $\sigma\left( \upsilon\right)$ denotes the absorption cross-section of alkali-metal atoms for photons, which is a function of the optical frequency $\upsilon$.

Meanwhile, the optical depth ($\mathrm{OD}$) of a cell with length $l$ can be defined as:

$$\begin{aligned} \mathrm{OD}=n\sigma\left( \upsilon\right)l\#\left( S4 \right) \end{aligned}$$

Consequently, the optical intensity exiting the cell can be expressed as $I(0)e^{-\mathrm{OD}}$.

Since circularly polarized light exerts a polarizing effect on alkali-metal atoms, the polarized alkali-metal atoms exhibit no absorption of circularly polarized radiation. The variation in optical intensity is described by the following expression:

$$\begin{aligned} \frac{d}{dz}I\left( z \right)=-n\sigma\left( \upsilon\right)I\left( z \right)\left( 1-P\left( z \right) \right)\#\left( S5 \right) \end{aligned}$$

Where $P\left( z \right)$ denotes the spin polarization rate of the alkali-metal atomic ensemble at propagation distance $z$ along the z-axis. Since the **Equation (S5)** constitutes a transcendental equation, it admits no closed-form general solution:

$$\begin{aligned} I\left( z \right)\exp\left( \frac{\sigma\left( \upsilon\right)I\left( z \right)}{R_{rel}} \right)=I\left( 0 \right)\exp\left( \frac{\sigma\left( \upsilon\right)I\left( 0 \right)}{R_{rel}}-n\sigma\left( \upsilon\right)z \right)\#\left( S6 \right) \end{aligned}$$

By utilizing the Lambert W-function, the form can be transformed into:

$$\begin{aligned} I\left( z \right)=\frac{R_{rel}}{\sigma\left( \upsilon\right)}W\left[ \frac{\sigma\left( \upsilon\right)I\left( 0 \right)}{R_{rel}}\exp\left( \frac{\sigma\left( \upsilon\right)I\left( 0 \right)}{R_{rel}}-n\sigma\left( \upsilon\right)z \right) \right]\#\left( S7 \right) \end{aligned}$$

Here, the Lambert W-function satisfies the relationship $W\left( z \right)e^{W\left( z \right)}=z$.

When the cell contains sufficient buffer gas and quenching gas, the equilibrium spin polarization rate of alkali-metal atoms within the cell is given by:

$$\begin{aligned} P_{0}=\frac{R_{op}}{R_{op}+R_{rel}}\#\left( S8 \right) \end{aligned}$$

The relationship between the optical pumping rate $R_{op}$ and the intensity $I$ of the pumping light can be expressed as:

$$\begin{aligned} R_{op}=\frac{I\sigma\left( \upsilon\right)}{Ah\upsilon}\#\left( S9 \right) \end{aligned}$$

Where $A$ is the cross-sectional area of the pumping light, and $h$ represents the Planck constant.

Thus, from the **Equations (S7)**, **(S8)** and **(S9)**, it follows that an optical intensity gradient of the pumping light exists within the cell. This induces a gradient in the pumping rate, which ultimately results in a spatial gradient in the spin polarization rate of the alkali-metal atoms along the propagation axis.

The pump beam propagating along the z-axis exhibits a transverse Gaussian intensity profile in the x-y plane. The optical pumping rate $R_{op}$ can be expressed as:

$$\begin{aligned} R_{op}\left( x,y,z \right)=R_{op}\left( z \right)\exp\left[ \frac{-2\left( x^{2}+y^{2} \right)}{r^{2}} \right]\#\left( S10 \right) \end{aligned}$$

Here, $r$ denotes the beam radius of the pumping light.

Within the conventional unidirectional OPAM architecture, the intensity variation of the forward-propagating right circularly polarized light traversing the cell can be described as:

$$\begin{aligned} \frac{d}{dz}I_{1}\left( z \right)=-n\sigma\left( \upsilon\right)I_{1}\left( z \right)\left( 1-P\left( z \right) \right)\#\left( S11 \right) \end{aligned}$$

The numerical simulation result for the spin polarization based on the conventional unidirectional system is depicted in **Figure S1a**. With an incident beam spot diameter of 2 mm and a propagation distance of 5 mm within the ^87^Rb cell, it is observed that the spin polarization rate remains high at the front section of the cell but undergoes rapid attenuation toward the rear section along the z-axis propagation path. This results in non-uniform spin polarization across the alkali-metal atomic ensemble, exhibiting a pronounced polarization gradient. Such significant polarization gradient directly acts upon the alkali-metal atomic ensemble serving as the sensing core, thereby inducing spatial differences in the relaxation characteristics of the ^87^Rb atomic ensemble. This phenomenon fundamentally compromises the operational stability and fundamental sensitivity limit of the OPAM.

Within the proposed polarization-improved bidirectional-pump OPAM system, the backward-propagating left circularly polarized light exhibits the following propagation dynamics through the cell:

$$\begin{aligned} \frac{d}{dz}I_{2}\left( z \right)=n\sigma\left( \upsilon\right)I_{2}\left( z \right)\left( 1-P\left( z \right) \right)\#\left( S12 \right) \end{aligned}$$

The simulation result for the bidirectional-pump system is presented in **Figure S1b**. The counterpropagating right and left circularly polarized beams incident along the z-axis possess equal intensities, each matching the intensity of a unidirectional incident beam. Due to the bidirectional-pump architecture, the intensity attenuation of each beam along the z-axis is effectively compensated by the opposing beam. Consequently, the gradient of the spin polarization rate along the z-axis within the cell is significantly mitigated compared to unidirectional illumination. This spatially uniform polarization of the alkali-metal atomic ensemble enhances the operational stability of the OPAM system. Furthermore, the system exhibits an enhancement in overall performance, achieving an improved fundamental sensitivity limit.

**
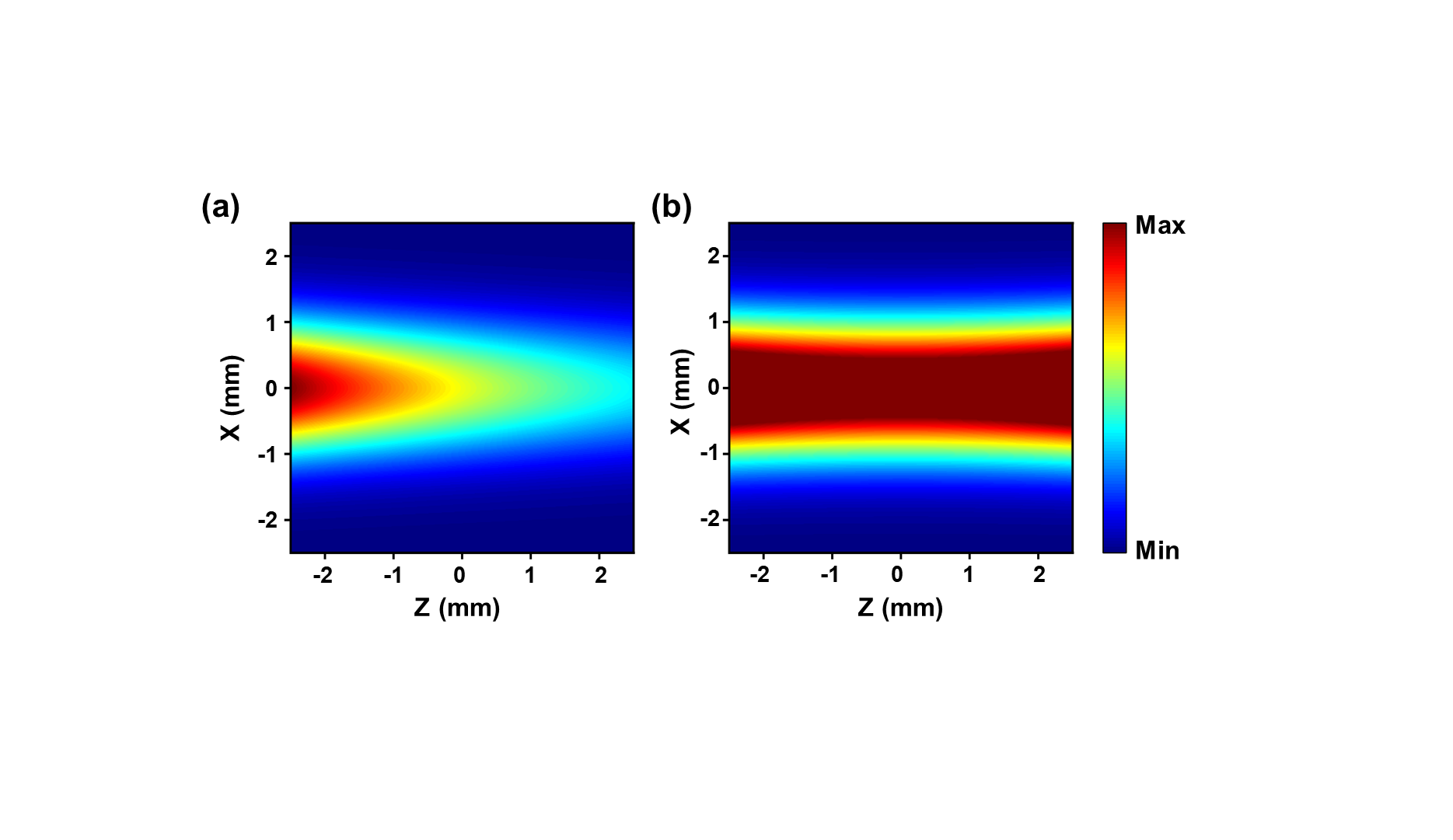
**

**Figure S1.** The numerical simulation for the spin polarization of atomics. a) The numerical simulation result for the spin polarization of atomics based on the conventional unidirectional system. b) The numerical simulation result for the spin polarization of atomics based on the bidirectional-pump system.

**Note 2. The sensitivity of the system constructed using a laser with a 1 MHz bandwidth.**

Given the substantial impact of the bandwidth of different pumping lasers on the polarization gradient of alkali-metal atoms, to demonstrate the universality of our design scheme, we constructed three systems using lasers with a bandwidth of 1 MHz. By adjusting the intensity of the incident light, the optical power in each system is maintained at 1 mW before entering the cell, consistent with prior experimental configurations. All parameters are set consistently, and the sensitivities of each system are presented in **Table S1**.

**Table S1. The sensitivity of the system constructed using a laser with a 1 MHz bandwidth**.

| **System Type** | **Sensitivity @10 Hz (pT/Hz^1/2^)** |
| --- | --- |
| detuning by double lasers | 5.76 |
| single pump by single-laser | 11.36 |
| double pump by single laser (this work) | 2.07 |

**Note 3.** **The influence of system fluctuations on sensitivity.**


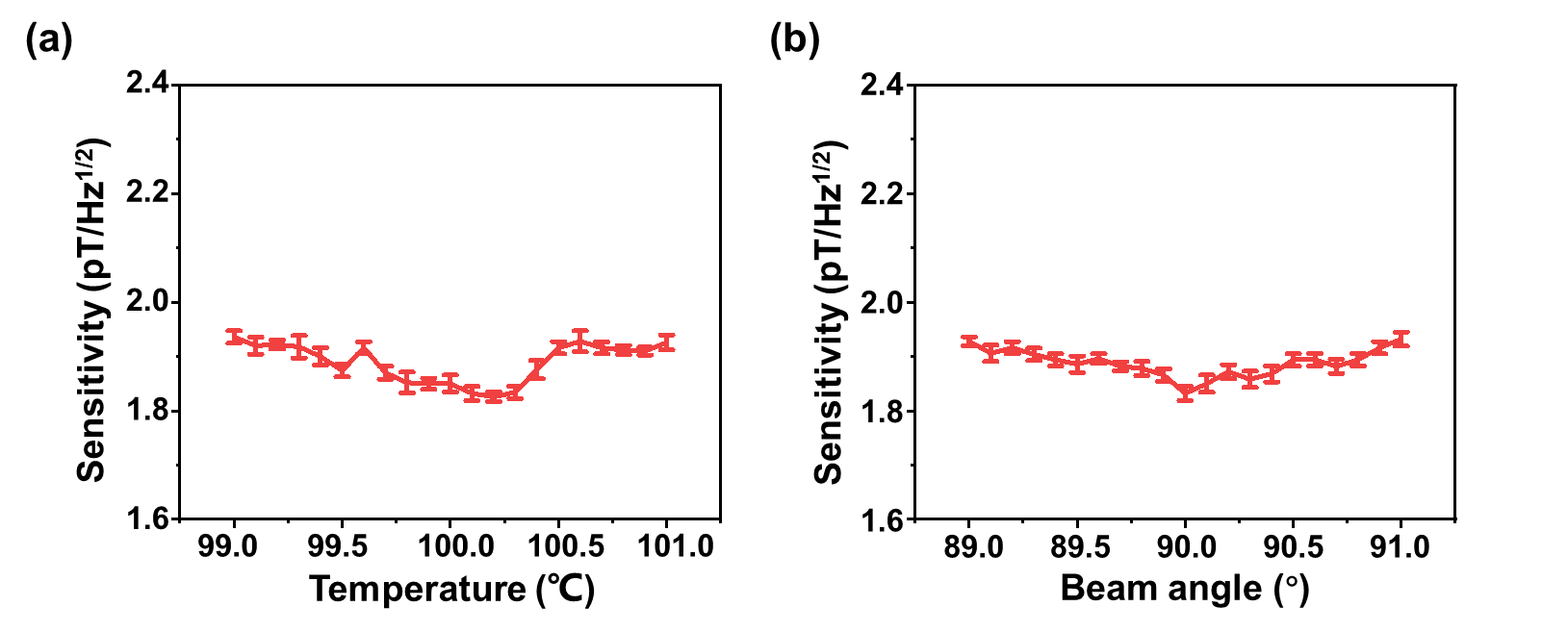


**Figure S2.** The influence of system fluctuations on sensitivity. a) The sensitivity of the system under different artificially set temperature fluctuations (measured 5 times for each temperature). b) The sensitivity of the system under different artificially set double-beam included angles (measured 5 times for each angle).

We examined the impacts of temperature variations and the orthogonality of the two beams on the system's sensitivity.

We employ the digital temperature controller (TCM-M207) to regulate the heating temperature of the cell with a control precision of 0.01 ℃, and the fluctuation error remains within ±0.1 ℃. The ideal heating temperature is set at 100℃. To investigate the effects of heating temperature errors and temperature fluctuations on the system, we artificially set the heating temperature between 99 ℃ and 100 ℃, adjusting it in increments of 0.1 ℃. We conducted five measurements at each temperature to verify the stability of the system. As depicted in **Figure S2a**, the sensitivity exhibited no significant change, thereby demonstrating that the temperature fluctuations resulting from errors have negligible impact on the system.

We also investigated the influence of the orthogonality of the two beams in the system on the system's sensitivity. During the calibration of the position and angle of the reflecting mirror, a six-axis displacement stage (L90A65S-L6, LBTEK) was employed for precise adjustment to ensure the orthogonality of the two beams. The control range of the displacement stage is as follows: the linear travel along the X, Y, and Z axes is 25 mm, while the rotational travel for $\theta_{X}$, $\theta_{Y}$, and $\theta_{Z}$ is ±8°, ±10°, and 360°, respectively. This is sufficient to satisfy the requirements for system adjustment. To verify the influence of beam misalignment on the system's sensitivity, we artificially adjusted the angles of the two beams to range from 89° to 91°, setting a sampling point at intervals of 0.1° to simulate angular errors. The system's sensitivity was measured five times at each angle, as depicted in **Figure S2b**. A slightly non-orthogonal angle may exert a certain influence on the system's sensitivity. However, within ±1°, the sensitivity fluctuation remains within 5%. This suggests that the angular error of the system's light is unlikely to have a substantial impact on the system's sensitivity.

**Note 4. The sensitivity of the system constructed using a cell consisting of ^39^K alkali-metal atoms.**

The bidirectional-pump system designed based on the spin-decoupled metasurface can effectively suppress the atomic polarization gradient, thereby significantly improving the system's sensitivity, and exhibits excellent universality across various atomic magnetometer. We utilized a ^39^K alkali-metal cell. A 770 nm laser, featuring a bandwidth of 500 kHz, was employed as the pump light and resonated with the D1 spectral line of the ^39^K atoms. By maintaining all other parameters consistent, we constructed the classic detuning by double lasers system, the single pump by single laser system, as well as the double pump by single laser system that we designed. The sensitivities of these three systems were compared, as presented in **Table S2**. The sensitivity of the system has been enhanced from 6.07 pT/Hz^1/2^ to 1.99 pT/Hz^1/2^.

**Table S2.** **The sensitivity of the system constructed using a cell consisting of ^39^K alkali-metal atoms**.

| **System Type** | **Sensitivity @10 Hz (pT/Hz^1/2^)** |
| --- | --- |
| detuning by double lasers | 6.07 |
| single pump by single-laser | 12.44 |
| double pump by single laser (this work) | 1.99 |
